# Exploitation of host mating behaviour by a parasitoid fly

**DOI:** 10.1098/rsbl.2026.0224

**Published:** 2026-10-21

**Authors:** Jacques Brodeur, Josée Doyon, Gabriele Rondoni, Simon Legault

**Affiliations:** ^1^ Institut de recherche en biologie végétale, Département de sciences biologiques, Université de Montréal, 4101 rue Sherbrooke E., Montréal, Québec, H1X 2B2, Canada; ^2^ Department of Agricultural, Food and Environmental Sciences, University of Perugia, 16123 Perugia, Italy

**Keywords:** *Istocheta aldrichi*, parasitoid fly, *Popillia japonica*, Japanese beetle, parasitism, host selection

## Abstract

Egg-laying decisions are crucial in insect parasitoids because the host constitutes the only resource available for immature development. We estimated the extent to which host sex, size and mating behaviour are involved and interact in shaping host selection in the association between the Japanese beetle, *Popillia japonica*, and a parasitoid fly, *Istocheta aldrichi*. Under field conditions, we found that females and larger individuals of the Japanese beetle, both males and females, are associated with the highest levels of parasitism, regardless of seasonal variations in host size and sex ratio. Furthermore, we propose that foraging *I. aldrichi* females exploit the sexual selection behaviour of the host to their own advantage. The increased vulnerability to parasitism of Japanese beetle females during mating when immobilized by males, the extended duration of mating and the preference of males for larger females contribute to the strong bias towards large females being the most parasitized. This leads to the hypothesis that *I. aldrichi* females have evolved to ‘merely’ rely on Japanese beetle males to select the most suitable hosts for reproduction. Finally, we discuss the possible impact on both seasonal and long-term demographics of the Japanese beetle populations and biological control.

## 1. Introduction

Animals are expected to select resources that maximize their lifetime fitness. For insect parasitoids, host selection is of critical importance because the host is the only resource available to the immature during its development [1,2]. Potential hosts vary in availability, detectability and suitability [3], and a variety of host-related determinants such as species, developmental stage, age, colour, sex, size, density, immune response and behavioural defence mechanisms affect the dynamic expression of host use by parasitoids [1]. Oviposition decisions can further be modulated by the physiological and informational state of the foraging female (e.g. life expectancy, egg load, previous experience, perception of the environment) [4–6]. To understand egg-laying decisions in parasitoid species, it is important to estimate the extent to which the above determinants are involved and how they interact.

Because of its significance in determining parasitoid fitness, host size is a trait that often has the greatest influence on parasitoid oviposition decisions. This is related to the adult size-fitness hypothesis, that is, individual fitness increases with increasing size [7]. Larger hosts contain higher amounts of nutrients for developing parasitoids, which generally confers physiological and behavioural benefits to adults, mainly in terms of fecundity, longevity, foraging capacity and mate acquisition.

Sexual size dimorphism has been reported in most animal species [8]. Among insects, females are generally larger than males, reflecting differences in their reproductive roles and in the underlying physiological mechanisms [9]. Unlike most hymenopteran parasitoids, several groups of dipteran parasitoids have succeeded in exploiting adult insect hosts (e.g. Phoridae, Tachinidae) [10]. Among these, females preferentially lay eggs on either males or females, or do not show preference between sexes. In most documented cases, sex-biased host selection is determined by behavioural events leading to oviposition, rather than by differences in suitability between male and female hosts. For example, the use of aggregation pheromone or mating calls (phonotaxis) by foraging parasitoid females may result in significant sex-biased parasitism [10].

In this study, we determined how host sex and size influence levels of parasitism in the association between the Japanese beetle, *Popillia japonica* Newman (Coleoptera: Scarabaeidae) and a parasitoid fly, *Istocheta aldrichi* (Mesnil) (Diptera: Tachinidae) (figure 1a). Next, we considered how the host's mating behaviour may be used for the parasitoid's benefit. Following the initial observations of Clausen *et al*. [11] (see §2a), we suggest there is an evolutionary dimension to the determining role of host mating in shaping host selection by *I. aldrichi*.

**Figure 1.**
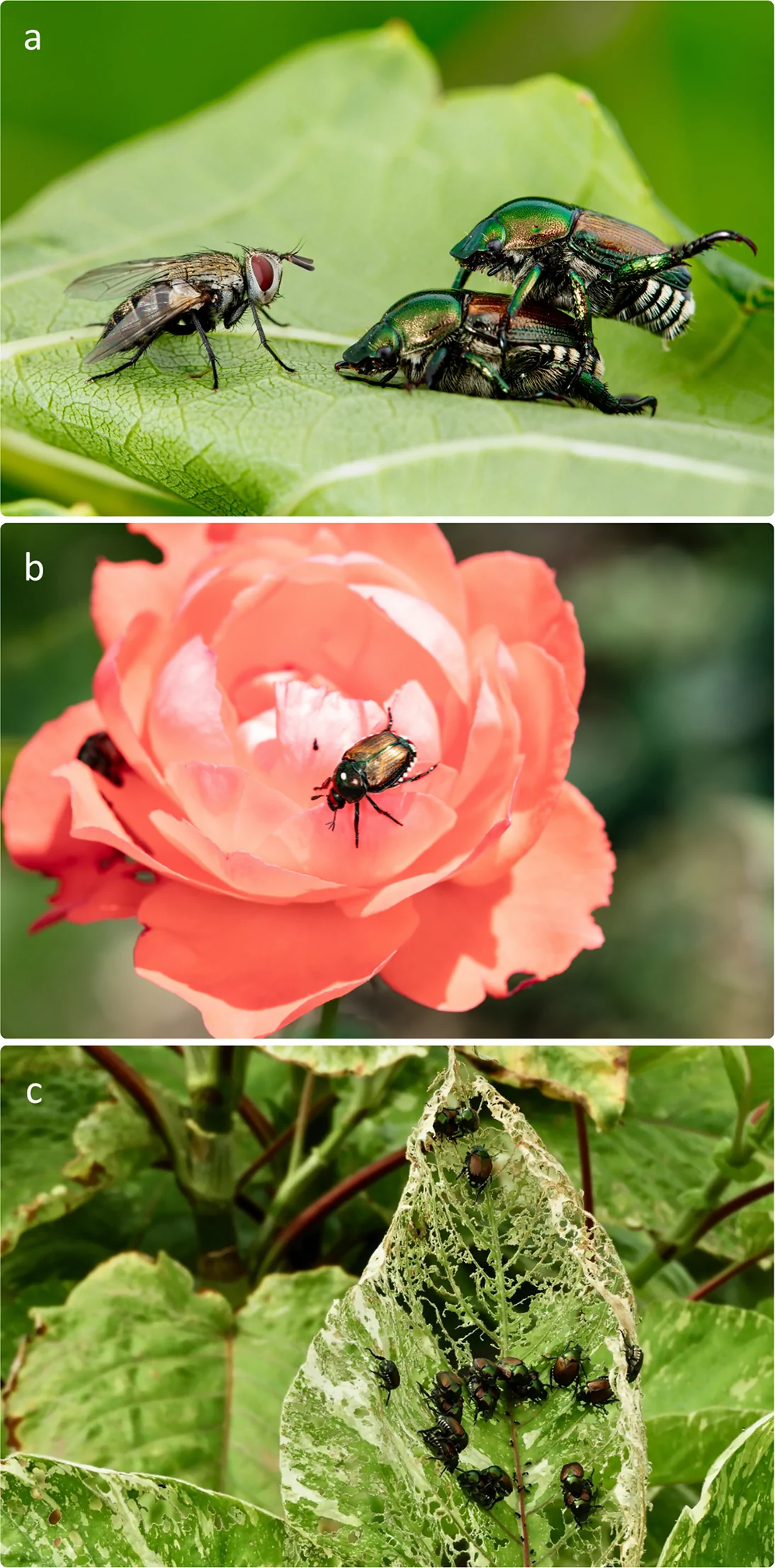
(a) An *I. aldrichi* female facing a Japanese beetle couple. The male beetle exhibits both mate guarding by firmly grasping the female and a defensive posture by raising its hind legs in front of the tachinid fly. Photo by J. Moisan-De Serres. (b) A Japanese beetle feeding on a rose. The individual had been parasitized by *I. aldrichi*, as seen by the large, and white egg laid on the host pronotum. Photo by J. Moisan-De Serres. (c) Aggregation of Japanese beetles feeding and mating on a Japanese knotweed leaf. Photo by J. Brodeur.

## 2. Material and methods

### (a) Study system

The Japanese beetle is a gregarious and polyphagous herbivore of economic importance [12,13]. Native to Japan, this invasive pest established in North America in the early 1910s [14] and more recently in Europe [15]. The Japanese beetle mating system is characterized by contest competition (formerly termed scrambled competition by Kruse & Switzer [16] and Kelly [17]), with males (polygyny) and females (polyandry) having multiple partners [18]. Virgin females emit a sex pheromone to attract males [19,20]. Once mated, females no longer release sex pheromone [20], and subsequent pairing occurs on food plants [16]. Males and females are then attracted to plant volatiles released by damaged leaves [20], resulting in the formation of large clusters of beetles, up to thousands of individuals, which provide mating opportunities, as suggested by Loughrin *et al.* [21].

The insect exhibits sperm precedence (the last male to copulate with a female fertilizes most of the eggs [22]) and post-copulatory mate guarding may further reduce sperm competition. Copulation lasts for an average of 132.5 seconds, whereas the duration of post-copulatory behaviour is highly variable, from a few minutes to over 24 hours [18,23].

*Istocheta aldrichi* is a solitary parasitoid of adults of the Japanese beetle, its only known host, based on existing documented Tachinidae–host associations [24]. It was imported to the United States in the 1920s as a promising biological control agent of the Japanese beetle [11,25]. Populations became established and spread naturally or were redistributed in northeastern America [24–26].

The Japanese beetle–*I. aldrichi* association constitutes an ideal model system to investigate parasitoid oviposition determinants and the demographic outcomes for the host. First, both insects have become abundant in our study area [26] and can be captured in large numbers using commercial traps [27]. Second, parasitism by *I. aldrichi* can easily be quantified because large white eggs are clearly visible to the observer (figure 1b) and mostly glued to the pronotum of the host (>95%) [28]. Third, the pioneer study of Clausen *et al*. [11] and a few more recent ones provide valuable information about oviposition determinants in *I. aldrichi*. Females are more susceptible to parasitism than males [11,27,29], and large hosts are parasitized to a greater extent than smaller hosts [27]. Furthermore, although not quantified, *I. aldrichi* oviposition typically occurs during host mating [11].

### (b) Japanese beetle abundance and parasitism rates

Insects were sampled in Maisonneuve Park located in Montréal, Canada, over a 6-year period (2019–2024). Insects were captured using one Bioprotec trap baited with a double lure system comprising a blend of floral compounds [30] and the *P. japonica* sex pheromone [19]. These traps are highly effective in capturing Japanese beetle males and females and provide reliable estimates of life-history traits and population parameters (e.g. size, sex ratio, parasitism rate by *I. aldrichi*) [27]. The trap was installed at the top of a 90-cm U-shaped bamboo stick. Sampling was conducted from late June to October to cover the entire period of adult Japanese beetle activity [26]. The contents of the trap were collected twice a week, and samples were next stored at −20°C.

For each sample, all beetles were counted to determine abundance and examined under a stereomicroscope for the presence of *I. aldrichi* eggs to estimate parasitism rate (i.e. number of beetles bearing at least one parasitoid egg/total number of beetles).

### (c) Sex-specific Japanese beetle abundance and parasitism rate

To quantify seasonal variations in sex-specific Japanese beetle abundance and parasitism rate, each year we selected several sampling periods (i.e. trap sampling dates) across the entire season (7.5 periods per year with a 13.1-day interval between them, on average). A subsample of 200 beetles (or all beetles when *n* < 200, mostly during early and late periods; see figure 2a) was used to estimate the proportion of each sex. Beetles were examined under a stereomicroscope to determine their sex using foreleg morphology, males being distinguishable from females by their pointier tibial spurs [31]. Sex ratios were used to estimate sex-specific Japanese beetle abundance and sex-specific parasitism rate.

**Figure 2.**
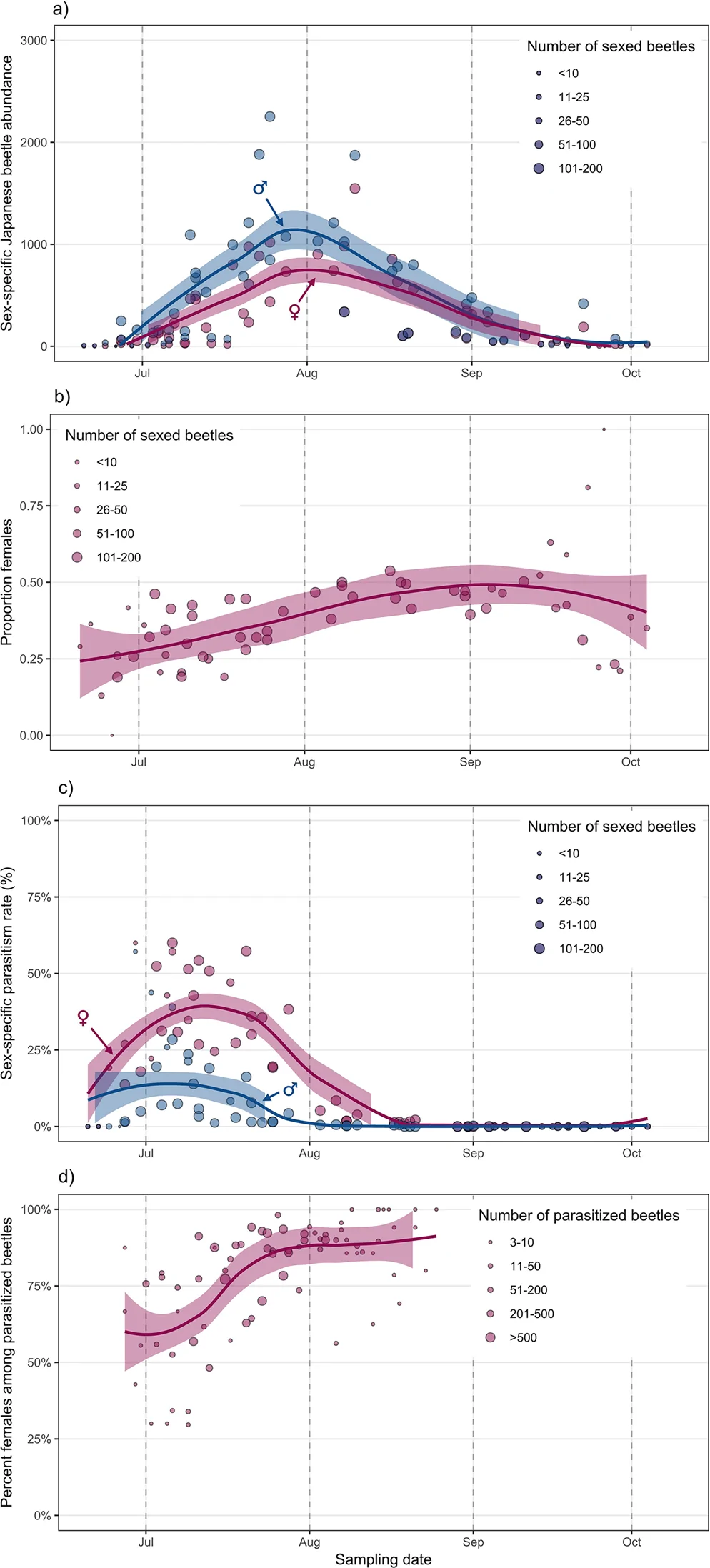
(a) Sex-specific Japanese beetle abundance, (b) proportion of female Japanese beetles, (c) sex-specific parasitism rates by *I. aldrichi* and (d) percentage of females among parasitized beetles. Size of dots is proportional to the number of beetles used to estimate each parameter.

### (d) Japanese beetle size distribution and size-specific parasitism rate

Subsamples of 30 beetles of each sex (or all beetles when *n* < 30, mostly during early and late periods; see figure 3a) were chosen randomly from selected periods for right elytral width measurements. This morphological trait is positively correlated with body and wing length in both sexes [17]. Beetles were placed individually in a 3-cm Petri dish filled with fine sand for support [17], oriented perpendicularly to the lens of a stereo microscope, and photographed at 7.5× magnification using a Leica FLEXACAM C1 camera. The right elytral width was measured to the nearest 0.01 mm using ImageJ software [32] (see [27] for a figure, indicating between which points the measurement was taken).

**Figure 3.**
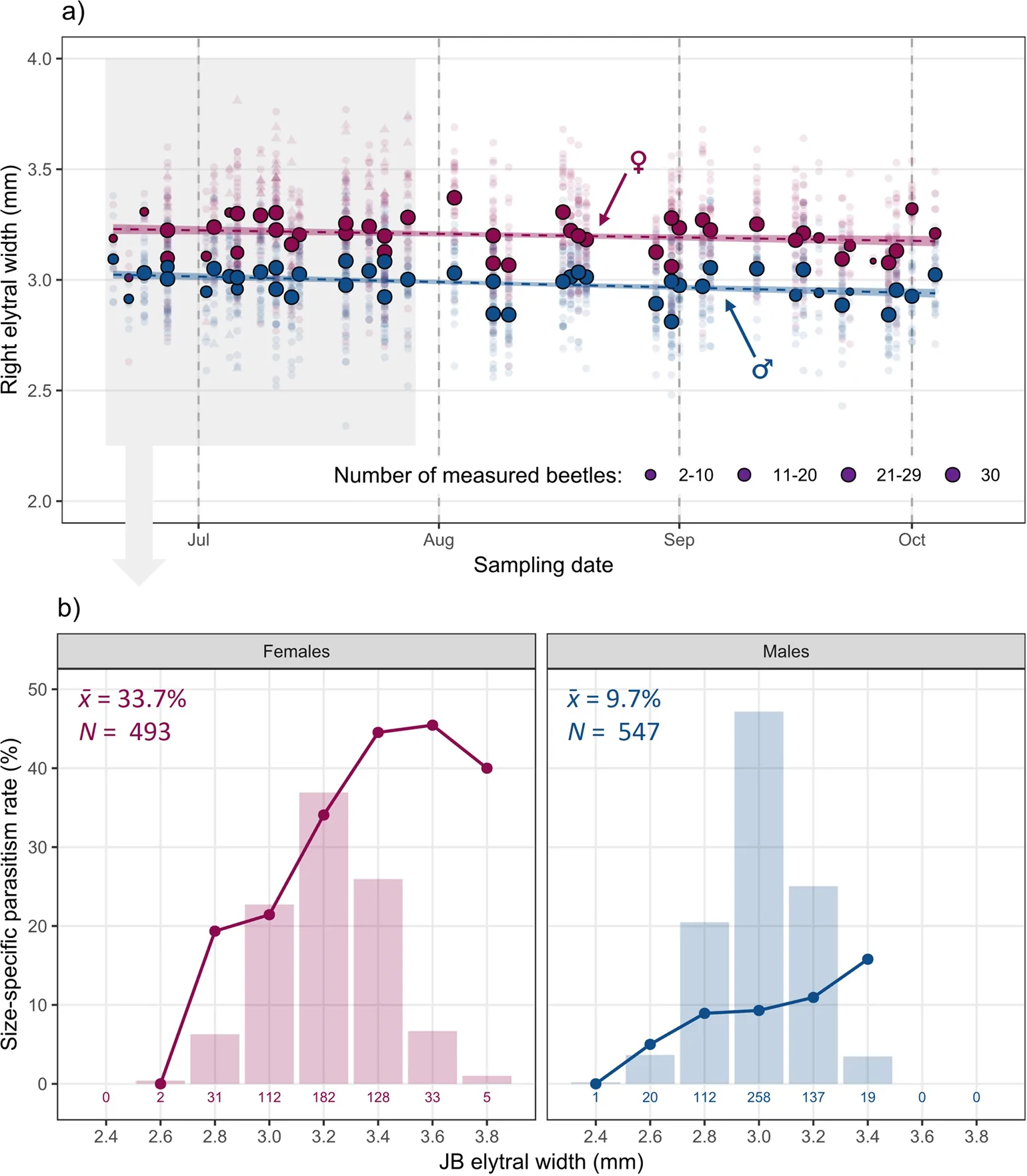
(a) Seasonal variations in Japanese beetle size distributions from 2019 to 2024 (*n* = 2345). Smaller, shaded dots represent individual measurements, whereas larger dots indicate the mean values for each sex and sampling date, their size being proportional to sample sizes. The shaded grey box corresponds to samples analysed in the bottom panel. (b) Host size-specific parasitism rates (%) by *I. aldrichi* (solid lines). Shaded bars show the frequency (%) distribution of host size classes per sex, and numbers below bars indicate sample sizes. *x̄* values indicate parasitism rates across all size classes.

Size-specific parasitism was evaluated for beetles captured in June and July of each year, which corresponds to the period of activity of *I. aldrichi* [26]. We first established the size distribution of male and female beetles according to eight different size classes (class elytral width = 0.2 mm), and then calculated the corresponding sex-specific parasitism rates by dividing the number of parasitized beetles measured by the total number of individuals.

### (e) Statistical analyses

Sex-specific Japanese beetle parasitism rates estimated from the complete dataset (total of 41 213 individuals over the 6-year period, from which 8639 beetles were sexed) were compared using a test for equality of proportions with continuity correction (*prop.test* function) in R [33]. The effects of Japanese beetle sex, sampling period and their interaction on right elytral widths, estimated for selected periods (a total of 2345 individuals were sized over the 6-year period), were evaluated using a linear mixed model (LMM) with years as a random factor. The model was implemented as in the R function *lmer* (*lme4* package [34]). Elytral width values satisfied the assumption of normality and homoscedasticity. The effects of Japanese beetle sex, size class and their interaction on size-specific parasitism rates were evaluated using a GLMM with a binomial error structure, with years as a random factor. The model was implemented as in the R function g*lmer* (*lme4* package [34]). In both models, numerical explanatory variables were standardized to zero mean and unit variance.

## 3. Results

### (a) Sex-specific Japanese beetle abundance and parasitism rate

The seasonal abundance of Japanese beetle adults was unimodal over the 2019–2024 period (figure 2a). Beetles were captured in traps as early as 24 June, and their number consistently increased before peaking around late July to early August. Abundance then declined gradually until early October. Males started to emerge earlier than females (protandry) and remained more abundant until the end of the season (figure 2a), with an overall sex ratio of 0.45 (proportion of females; figure 2b).

On average, parasitism was maximal around mid-July and declined gradually until mid-August. Total seasonal parasitism was significantly higher for females (14.4%, *n* = 16 345) than for males (3.6%, *n* = 24 868; *X*^2^ = 2294.7, df = 1, *p* < 0.001). This pattern was consistent throughout the season (figure 2c), and the difference in parasitism between sexes was maximum when Japanese beetle populations were increasing (early July) and potential hosts became abundant (figure 2a). Overall, 81.2% of parasitized beetles were females (*n* = 9217; figure 2d).

### (b) Japanese beetle size distribution and size-specific parasitism rates

On the basis of elytral width measurements, females were significantly larger than males (3.20 ± 0.03 mm (*n* = 1148) vs. 2.98 ± 0.03 mm (*n* = 1197)), and for both sexes, adult size slightly decreased over the season (table 1, figure 3a). We found no significant interaction between beetle sex and sampling period (table 1). During the activity period of *I. aldrichi* (late June and July), size-specific parasitism rates were significantly higher for females, and increased with increasing size class for both sexes, reaching 40%–45% in larger females (figure 3b, table 2).

**Table 1.** Summary of the LMM for the effects of sex, sampling period and their interaction on adult size (right elytral width) of Japanese beetles collected in 2019–2024.

variables	coefficient	±	s.e.	*T*	*p*	*X^2^*	*df*	*p*
—	—		—	—	—	859.37	3	<0.001
males vs. females	−0.2220	±	0.0070	−31.690	<0.001			
sampling date	−0.0205	±	0.0051	−4.002	<0.001			
interaction	−0.0069	±	0.0070	−0.981	0.327			

**Note:** *X*^2^ value and the corresponding *p*-value relate to the entire model.

**Table 2.** Summary of the generalized LMM for the effects of sex, size class and their interaction on size-specific parasitism rates of Japanese beetles collected in June and July 2019–2024.

variables	coefficient	±	s.e.	*Z*	*p*	*X^2^*	*df*	*p*
—	—		—	—	—	89.303	3	<0.001
males vs. females	−0.8694	±	0.1867	−4.657	<0.001			
size class	0.7860	±	0.2109	3.726	<0.001			
interaction	0.2117	±	0.4458	0.475	0.635			

**Note:** *X*^2^ and the corresponding *P*-value relate to the entire model.

## 4. Discussion

We explored the role of host sex and size in the oviposition behaviour of a parasitic fly under field conditions. Consistent with previous studies, we found that females, as well as larger males and females of the Japanese beetle individuals, are associated with the highest levels of parasitism by *I. aldrichi*. Here, we first examine the significance of this egg-laying pattern on *I. aldrichi* reproductive success. We next present an original perspective, suggesting that an evolutionary scenario underlies the determining role of host mating in shaping host exploitation by *I. aldrichi*. Finally, we point out the possible impact on both seasonal and long-term demographics of the Japanese beetle populations and biological control.

### (a) A matter of host sex

Ovipositing *I. aldrichi* females mostly target Japanese beetle females that account for 81.2% of all parasitized individuals. This value is within the range of previous data for native populations in Japan (80%–96% [11]), and for established populations in the USA and Canada: Connecticut (79% [35]), Minnesota (80% [29]) and Ontario (81% [36]). Furthermore, when occurrences of super-parasitism (more than one egg per host) are included in our analysis, 95.9% of all parasitoid eggs were deposited on females (S. Aubé 2026, unpublished data). Although Japanese beetles’ sex ratio was male-biased throughout the season, parasitism was always higher for females than for males.

Differences in host sex suitability are not probably to contribute to the observed parasitism bias because, for a given size, both sexes are equally able to sustain the development of *I. aldrichi* (J.D. 2026, unpublished data). Such sex-biased parasitism arises from both body size differences between sexes and host mating behaviour, which we explore below.

### (b) A matter of host size

Japanese beetle females are generally larger than males, a pattern that persisted throughout the season in our study. Larger Japanese beetle hosts are associated with the highest levels of parasitism by *I. aldrichi*, regardless of their sex, as also reported by Legault *et al.* [27]. This relationship concurs with the adult size-fitness hypothesis [7]. Accordingly, there is a strong, telescopic linear relationship between host size, parasitoid puparium weight and parasitoid adult size in *I. aldrichi* (J.D. 2026, unpublished data).

### (c) A matter of host mating

Several components of the Japanese beetle sexual selection behaviour together shape the oviposition pattern in *I. aldrichi* and render larger females the most vulnerable to parasitism. The key elements to consider are (i) host pairing, because of its attractivity to the parasitoid female; (ii) the intrinsic vulnerability of the host female during copulation and mate guarding; and (iii) the mating preference of Japanese beetle males for larger females.

First, common features of Japanese beetle aggregation on a host plant are the substantial number of individuals of both sexes and the high frequency of mating events [21,37]. This is illustrated in figure 1c, where 19 beetles are observed on a Japanese knotweed leaf, with 14 of them forming 7 couples. A recent study described how *I. aldrichi* females locate Japanese beetle aggregations in the environment, and more specifically, mating couples on infested plants (G.R. *et al*. 2026, unpublished data). Olfactometer bioassays showed that, at long range, parasitoid females are attracted to plant-feeding damage, whereas, at close range, they respond more to the odour of Japanese beetle couples than to that of solitary males or females. These results provide a mechanistic interpretation of the initial observation by Clausen *et al*. [11], who reported that *I. aldrichi* oviposition mostly occurs during host mating.

Second, our study reinforces Clausen *et al*. [11], who introduced an explanation for the sex-biased parasitism in the Japanese beetle: *I. aldrichi* females lay their eggs on females of mating pairs because male guarding would prevent the defensive behaviour of female beetles when threatened. Individuals of both sexes have the capacity to fend off an attack by natural enemies by dropping from a leaf or raising their hind legs (figure 1a). However, when grabbed firmly by males, by both legs and genitalia during copulation, and only legs during mate guarding [23], Japanese beetle females become highly vulnerable to an attack by *I. aldrichi* females (see Pelletier *et al.* [28] for a link to a video file). The extended duration of copulation and mate-guarding prolongs the window of Japanese beetle vulnerability to parasitism.

Third, Japanese beetle males prefer to pair with larger females because they produce more and larger eggs than smaller females [17,38]. This pattern would therefore also indirectly shape host selection by *I. aldrichi* because oviposition most probably occurs during host mating. This is true for females, as discussed above, but may also apply to males, because larger individuals are more frequently observed in mated pairs and are more successful at displacing smaller conspecifics for mating opportunities [16], with the provision that parasitism on males could also occur during mating, a possibility that remains to be explored.

Together, the response of parasitoid females to odours from Japanese beetle couples, the increased vulnerability of female hosts during mating, the extended duration of mating and the preference of host males for larger females contribute to the strong bias towards Japanese beetle females and larger individuals, both males and females, being the most parasitized by *I. aldrichi*. These observations lead us to hypothesize that host selection in *I. aldrichi* is mainly governed by the sexual selection behaviour of its host. Mating Japanese beetle couples are composed of the most suitable hosts for *I. aldrichi* reproduction. Under such a scenario, we suggest that *I. aldrichi* has adapted to exploit the gregarious and promiscuous (high frequency of mating events) behaviours of its host. As a result, host-seeking females probably do not have to make significant oviposition decisions with regard to host sex and size once they have targeted mating couples within Japanese beetle aggregations. The interaction between the Japanese beetle and *I. aldrichi* has potentially evolved into a situation where parasitoid females exploit host mating behaviour and male mate selection to select and render vulnerable the most suitable hosts for parasitoid immature development. This pattern represents a particularly spectacular example of the exploitation of a host mating system by a parasitoid.

### (d) Demographic consequences

For Japanese beetles, our results can be examined in the context of the mating-risk mortality hypothesis [39–41], which refers to an increased risk of parasitism for individuals engaging in copulation and mate guarding. Such a viability cost may influence the seasonal and long-term structure of Japanese beetle populations. For instance, to what extent could the decrease in adult size we observed in both sexes over the season be owing to the elimination of larger individuals by the parasitoid fly? Such a pattern could also modify the stage-structured populations through a decrease in sex ratio (proportion of females) and the number of more fecund individuals (large females), leading to a reduction in the intrinsic growth rate of the host population. Female-biased parasitism could also destabilize host–parasite dynamics and promote population breakdown [42]. Over longer time scales, sex- and size-biased parasitism by *I. aldrichi* could select for genetic changes with respect to life-history traits, including size, of the Japanese beetle. For example, overfishing reduces not only the abundance of fish in the ocean but also their body size through selection favouring smaller individuals [43]. The Japanese beetle–*I. aldrichi* association constitutes a promising system in which to explore these demographic dynamics, notably because the parasitoid is currently progressing naturally or being redistributed across new regions, possibly experiencing evolutionary changes that could be documented.

From a biological control perspective, the strong bias towards large females being the most parasitized hosts adds value to using *I. aldrichi* as a biological control agent for the Japanese beetle. This parasitoid species targets the very host individuals that have the greatest impact on the pest's population growth. To our knowledge, the consequences of sex-biased parasitism on pest population dynamics remain to be quantified and modelled. As noted by Wellington [44] several years ago, pest control ‘would be better served by methods that would selectively discriminate against reproductively superior members of a generation than by methods that merely increase mortality indiscriminately’.

## Supplementary Material



## Data Availability

Data regarding parasitism rates, host sex and host size are provided as supplementary material associated with this article. Supplementary material is available online [45].

## References

[1] Godfray HCJ . 1994 Parasitoids: behavioural and evolutionary ecology. Princeton, NJ: Princeton University Press, Princeton, NJ, USA.

[2] Brodeur J , Boivin G. 2004 Functional ecology of immature parasitoids. Annu. Rev. Entomol. 49, 27–49. (doi:10.1146/annurev.ento.49.061703.153618) 14651455

[3] Vet LEM , Dicke M. 1992 Ecology of infochemical use by natural enemies in a tritrophic context. Annu. Rev. Entomol. 37, 141–172. (doi:10.1146/annurev.en.37.010192.001041)

[4] Rosenheim JA , Rosen D. 1991 Foraging and oviposition decisions in the parasitoid *Aphytis lingnanensis*: distinguishing the influences of egg load and experience. J. Anim. Ecol. 60, 873–893. (doi:10.2307/5419)

[5] Roitberg BD , Sircom J, Roitberg CA, van Alphen JJM, Mangel M. 1993 Life expectancy and reproduction. Nature 364, 108. (doi:10.1038/364108a0) 8321281

[6] Hopper KR , Prager SM, Heimpel GE. 2013 Is parasitoid acceptance of different host species dynamic? Funct. Ecol. 27, 1201–1211. (doi:10.1111/1365-2435.12107)

[7] Jervis MA , Ellers J, Harvey JA. 2008 Resource acquisition, allocation, and utilization in parasitoid reproductive strategies. Annu. Rev. Entomol. 53, 361–385. (doi:10.1146/annurev.ento.53.103106.093433) 17877453

[8] Fairbairn DJ , Blanckenhorn WU, Szekely T. 2007 Sex, size and gender roles. New York, NY: Oxford University Press. (doi:10.1093/acprof:oso/9780199208784.001.0001)

[9] Stillwell RC , Blanckenhorn WU, Teder T, Davidowitz G, Fox CW. 2010 Sex differences in phenotypic plasticity affect variation in sexual size dimorphism in insects: from physiology to evolution. Annu. Rev. Entomol. 55, 227–245. (doi:10.1146/annurev-ento-112408-085500) PMC4760685 19728836

[10] Feener Jr DH , Brown BV. 1997 Diptera as parasitoids. Annu. Rev. Entomol. 42, 73–97. (doi:10.1146/annurev.ento.42.1.73) 15012308

[11] Clausen CP , King JL, Teranishi C. 1927 The parasites of Popillia japonica in Japan and Chosen (Korea) and their introduction into the United States. Bulletin 1429. Washington, DC: United States Department of Agriculture.

[12] Brodeur J , Doyon J, Abram PK, Parent JP. 2024*Popillia japon*ica Newman, Japanese beetle (Coleoptera: Scarabaeidae). In Biological control programmes in Canada 2013–2022 (eds Vankosky MA, Martel V), pp. 343–350. Wallingford, UK: CAB International. (doi:10.1079/9781800623279.0037)

[13] Althoff ER , Rice KB. 2022 Japanese beetle (Coleoptera: Scarabaeidae) invasion of North America: history, ecology, and management. J. Integr. Pest Manag. 13, 1–11. (doi:10.1093/jipm/pmab043)

[14] Fleming WE . 1972 Biology of the Japanese beetle. Technical Bulletin 1449. Washington, DC: United States Department of Agriculture.

[15] Strangi A *et al.* 2024 Tracing the dispersal route of the invasive Japanese beetle *Popillia japonica*. J. Pest Sci. 97, 613–629. (doi:10.1007/s10340-023-01653-1)

[16] Kruse KC , Switzer PV. 2007 Physical contests for females in the Japanese beetle, *Popillia japonica*. J. Insect Sci. 7, 34. (doi:10.1673/031.007.3401) PMC2999439 20233099

[17] Kelly CD . 2020 Size and shape assortative mating in Japanese beetles (*Popillia japonica*). Can. J. Zool. 98, 691–695. (doi:10.1139/cjz-2020-0062)

[18] Tigreros N , Switzer PV. 2009 Observations of multiple mating under field conditions for Japanese beetles (*Popillia japonica* Newman) (Coleoptera: Scarabaeidae). J. Kansas Entomol. Soc. 82, 151–159. (doi:10.2317/JKES707.12.1)

[19] Tumlinson J , Klein M, Doolittle R, Ladd TL, Proveaux AT. 1977 Identification of the female Japanese beetle sex pheromone: inhibition of male response by an enantiomer. Science 197, 789–792. (doi:10.1126/science.197.4305.789) 17790774

[20] Ladd Jr TL . 1970 Sex attraction in the Japanese beetle. J. Econ. Entomol. 63, 905–908. (doi:10.1093/jee/63.3.905) 5431684

[21] Loughrin JH , Potter DA, Hamilton-Kemp TR. 1995 Volatile compounds induced by herbivory act as aggregation kairomones for the Japanese beetle (*Popillia japonica* Newman). J. Chem. Ecol. 21, 1457–1467. (doi:10.1007/BF02035145) 24233676

[22] Simmons LW . 2002 Sperm competition and its evolutionary consequences in the insects. Princeton, NJ: Princeton University Press.

[23] Barrows EM , Gordh G. 1978 Sexual behavior in the Japanese beetle, *Popillia japonica*, and comparative notes on sexual behavior of other scarabs (Coleoptera: Scarabaeidae). Behav. Biol. 23, 341–354. (doi:10.1016/S0091-6773(78)91365-2)

[24] Makovetski V , Abram PK. 2024 *Istocheta aldrichi* (Mesnil) makes its biological control debut in British Columbia, Canada. Tachinid Times 37, 4–10.

[25] Fleming WE . 1968 Biological control of the Japanese beetle. Technical Bulletin 1383. Washington, DC: United States Department of Agriculture.

[26] Gagnon ME , Giroux M, Doyon J, Legault S, Brodeur J. 2023 The establishment of the interaction between the Japanese beetle, *Popillia japonica* (Coleoptera: Scarabaeidae) and the parasitoid *istocheta aldrichi* (Diptera: Tachinidae) in Québec, Canada. Can. Entomol. 155, e32. (doi:10.4039/tce.2023.22)

[27] Legault S , Doyon J, Brodeur J. 2024 Reliability of a commercial trap to estimate population parameters of Japanese beetles, *Popillia japonica*, and parasitism by *Istocheta aldrichi*. J. Pest Sci. 97, 575–583. (doi:10.1007/s10340-023-01666-w)

[28] Pelletier M , Legault S, Doyon J, Brodeur J. 2023 Where and why do females of the parasitic fly *Istocheta aldrichi* lay their eggs on the body of adult Japanese beetle? J. Insect Behav. 36, 308–317. (doi:10.1007/s10905-023-09841-8)

[29] Shanovich HN , Ribeiro AV, Koch RL. 2021 Seasonal abundance, defoliation, and parasitism of Japanese beetle (Coleoptera: Scarabaeidae) in two apple cultivars. J. Econ. Entomol. 114, 811–817. (doi:10.1093/jee/toaa315) 33503253

[30] Ladd TL , Klein MG. 1986 Japanese beetle (Coleoptera: Scarabaeidae) response to color traps baited with phenethyl propionate, eugenol and geraniol. J. Econ. Entomol. 79, 84–86. (doi:10.1093/jee/79.1.84)

[31] Smith LB , Hadley CH. 1926 The Japanese beetle. United States Department of Agriculture Circular 363. Washington, DC: Government Printing Office.

[32] Schneider CA , Rasband WS, Eliceiri KW. 2012 NIH image to ImageJ: 25 years of image analysis. Nat. Methods 9, 671–675. (doi:10.1038/nmeth.2089) PMC5554542 22930834

[33] R Core Team . 2022 R: a language and environment for statistical computing. Vienna, Austria: R Foundation for Statistical Computing. https://www.R-project.org.

[34] Bates D , Mächler M, Bolker B, Walker S. 2015 Fitting linear mixed-effects models using lme4. J. Stat. Softw. 67, 1–48. (doi:10.18637/jss.v067.i01)

[35] Kidd KA . 1993 Oviposition and parasitism of Japanese beetle, *Popillia japonica* Newman (Coleoptera: Scarabaeidae) by *Istocheta aldrichi* (Diptera: Tachinidae) with notes on storage of *I. aldrichi* puparia. Report of Activities, Biological Control Laboratory, North Carolina Department of Agriculture, pp. 47–55.

[36] O'Hara JE . 2014 New tachinid records for the United States and Canada. Tachinid Times 27, 34–40.

[37] Sara S , McCallen EB, Switzer PV. 2013 The spatial distribution of the Japanese beetle, *Popillia japonica*, in soybean fields. J. Insect Sci. 13, 1–9. (doi:10.1673/031.013.3601) PMC3738102 23895634

[38] Saeki Y , Kruse KC, Switzer PV. 2005 Male preference for large females and female reproductive condition in the Japanese beetle, *Popillia japonica* Newman (Coleoptera: Scarabaeidae). J. Kansas Entomol. Soc. 78, 13–19. (doi:10.2317/JKES-0312.14.1)

[39] Gwynne DT . 1989 Does copulation increase the risk of predation? Trends Ecol. Evol. 4, 54–56. (doi:10.1016/0169-5347(89)90144-4) 21227316

[40] Magnhagen C . 1991 Predation risk as a cost of reproduction. Trends Ecol. Evol. 6, 183–186. (doi:10.1016/0169-5347(91)90210-O) 21232452

[41] Kemp DJ . 2012 Costly copulation in the wild: mating increases the risk of parasitoid-mediated death in swarming locusts. Behav. Ecol. 23, 191–194. (doi:10.1093/beheco/arr173)

[42] Boukal DS , Berec L, Krivan V. 2008 Does sex-selective predation stabilize or destabilize predator–prey dynamics? PLoS ONE 3, e2687. (doi:10.1371/journal.pone.0002687) PMC2444021 18628951

[43] Jørgensen C *et al.* 2007 Managing evolving fish stocks. Science 318, 1247–1248. (doi:10.1126/science.1148089) 18033868

[44] Wellington WG . 1977 Returning the insect to insect ecology: some consequences for pest management. Environ. Entomol. 6, 1–8. (doi:10.1093/EE/6.1.1)

[45] Brodeur J , Doyon J, Rondoni G, Legault S. 2026 Supplementary material from: Exploitation of host mating behaviour by a parasitoid fly. Figshare. (doi:10.6084/m9.figshare.c.8680208) PMC13682938 42901536

